# Abundance and Population Decline Factors of Chrysopid Juveniles in Olive Groves and Adjacent Trees

**DOI:** 10.3390/insects10050134

**Published:** 2019-05-07

**Authors:** Rafael Alcalá Herrera, Mercedes Campos, Marina González-Salvadó, Francisca Ruano

**Affiliations:** 1 Plant Protection Group, Department of Environmental Protection, Estación Experimental del Zaidín (EEZ-CSIC), C/Profesor Albareda 1, 18008 Granada, Spain; mercedes.campos@eez.csic.es; 2 Department of Zoology, University of Granada, Campus de Fuentenueva s/n, 18071 Granada, Spain; marina93_@hotmail.com (M.G.-S.); fruano@ugr.es (F.R.)

**Keywords:** parasitoids, *Chrysoperla carnea* complex, ecological infrastructure, *Olea europaea*, *Pinus halepensis*, *Prunus dulcis*, *Quercus rotundifolia*

## Abstract

Numerous species of the family Chrysopidae, commonly found in agroecosystems, whose larvae predate on several pests of economic importance, are regarded as biological control agents. Their abundance and diversity are influenced by vegetation cover, although little is known about the effects of semi-natural habitats on their populations. The objective of this study is to gain a better understanding of the relationship between the trees in semi-natural habitats adjacent to olive groves, juvenile stages of the family Chrysopidae and factors influencing their population decline, which is crucial for an effective habitat management program aimed at conserving these important predators. Using cardboard band traps (eight per tree), the juvenile stages were collected from 25 almond, oak, olive and pine trees over a one-year sampling period. The population decline was caused by parasitoids (26.5%), predators (5.1%) and unknown factors (13.2%). In addition, chrysopids established in olive trees showed the lowest rate of parasitism. We identified ten chrysopid species that emerged from the juveniles collected from almond, oak, olive and pine trees, with a predominance of *Pseudomallada prasinus*. The chrysopid–parasitoid complex was composed of five species; *Baryscapus impeditus* (Eulophidae), which was the most abundant, was preferentially associated with *Chrysopa pallens*, *Chrysoperla lucasina* and *Chrysoperla mediterranea*.

## 1. Introduction

Of the many families of the Order Neuroptera, Chrysopidae attracted the most attention as compared to Coniopterygidae and Hemerobiidae [1], as numerous species belonging to the Chrysopidae family are regarded as biological control agents given their potential impact on pest populations in crops [2,3,4,5,6]. Larvae are active polyphagous predators of soft-bodied arthropods, such as aphids, whiteflies, thrips and mites, in addition to being widely distributed in agroecosystems [2,3,4,5,6].

Chrysopidae is the second most important family in terms of the number and diversity of species with 1423 valid species belonging to 82 genera [7]. *Chrysoperla carnea* (Stephens, 1836) *sensu lato*, which has been reared and released in crops around the world [8,9,10,11], is the species most commonly used in agricultural biological control programs [12]. There is evidence that *C. carnea* is a complex of at least 21 cryptic species [1,13,14]. Although some species are well defined with respect to morphological characteristics, habitats, courtship songs and molecular techniques, their taxonomy has not been fully resolved [15,16,17,18,19]. A recent review of the green lacewing showed that seven species belong to the *Chrysoperla* Steinmann, 1964 genus in the Iberian Peninsula and Balearic Island [1].

In previous studies 33 species of the Chrysopidae family were identified in olive groves, with the *Chrysoperla carnea* complex (Stephens, 1836) and the genus *Pseudomallada* Tsukaguchi, 1995 being particularly noteworthy [20,21,22,23]. The larval stages of these chrysopids are key predators of the three main pests in olive groves: *Prays oleae* (Bernard, 1788), *Saissetia oleae* (Olivier, 1791) and *Euphyllura olivina* (Costa, 1839) [3,20,24,25]. The use of green lacewings to improve biological pest control in olive groves has been evaluated [26]. McEwen et al. [27] attempted to attract *C. carnea* by spraying artificial honeydew [27], and another study has shown that a relationship exists between non-crop vegetation and green lacewing oviposition in olive groves [28]. Porcel et al. [29] also found that resident vegetation cover has a positive effect on green lacewings abundance and diversity in olive groves. However, the role of semi-natural habitats adjacent (bordering and around) to olive groves is poorly understood.

Chrysopid populations are regulated by predation (intraguild and cannibalism) and parasitism which are particularly harmful [30,31], and their development is also affected by abiotic conditions such as temperature, humidity and day length [32,33,34,35,36]. In fact, the eggs and larvae of *C. carnea s.l.* are attacked and killed by coccinellids, reduvids, carabids, spiders and ants [37,38,39,40,41], as well as by cannibalistic individuals from its own species [42]. The chrysopid–parasitoid complex is composed of species from the Orders Hymenoptera and Diptera, in addition to mites, fungae and certain viruses, which can affect all stages of chrysopid development, ranging from the egg and larva stages to adulthood [31,43,44,45,46,47]; some genera of the Order Hymenoptera, such as *Isodromus* Howard, 1887, *Baryscapus* Förster, 1856, *Helorus* Latreille, 1802 and *Gelis* Thunberg, 1827 are primary parasitoids of Chrysopidae, while others, such as *Perilampus* Latreille, 1809, *Dichrogaster* Doumerc, 1855, *Pteromalus* Swederus, 1795 and *Eupelmus* Dalman, 1820, are primary parasitoids of Chrysopidae and hyperparasitoids [47,48,49,50,51,52,53,54,55,56,57,58]. Other factors affecting larval mortality include abiotic conditions and the food resource availability. The impact of all these factors can vary according to the species of chrysopid and its habitat, which need to be accurately characterized when biological control is planned both for conservation purposes and through mass release of chrysopids [59].

Faced with natural enemies, chrysopids have developed defensive strategies and behaviours, such as nocturnal and twilight activity, cryptic, aposematic and disruptive coloration [60,61], stalked eggs [62,63,64], thanatosis [6], as well as segregation of foul-smelling substances produced by adults and toxic, crippling and disruptive substances secreted by larvae [6,61,65,66,67,68,69]. It has also been suggested that exogenous material on the backs of larvae of certain chrysopid genera (*Pseudomallada* and *Rexa* Navás, 1920) could act as a physical barrier against predators and parasitoids [62,69,70,71,72,73].

Given the generalist predatory behaviour and dispersive capacity of chrysopids, their populations in olive groves are influenced by the vegetation and natural habitats adjacent to the crop, where they can find alternative prey, pollen, nectar, as well as reproduction and refuge sites. Thus, spontaneous vegetation cover between the rows of olive trees has been reported to increase chrysopid abundance and diversity in the crop [29]. Additionally, tree species such as *Quercus rotundifolia* Lam., *Pinus halepensis* Mill. and *Prunus dulcis* (Mill.) D.A. Webb, which are an integral part of the olive grove landscape in Spain, are visited by chrysopids [3,21,74] and used as oviposition sites by different species [75]. Studies of their population dynamics in olive groves should therefore include the effect of adjacent vegetation, as research on chrysopid parasitism has, up to now, focused on different arboreal species and crops while neglecting activity in the surrounding landscape [45,47,49,51,58,76,77,78,79].

This study aims to assess the relationship between trees in semi-natural habitats adjacent to olive groves, the juvenile stages of the family Chrysopidae and population decline factors (parasitism, predation and unknown factors).

We expected (a) to collect chrysopid juveniles from all the tree species studied, from which adult chrysopids had previously been sampled [21], and (b) to record a medium to high chrysopid parasitism rate in olive trees which was predicted to be similar in all three trees species (almond, oak and pine) [50,78,80]. Finally, as we expected the chrysopids to be parasitized, we studied the relationship between parasitoid and chrysopid assemblages while taking into account the season and tree species (almond, oak, olive and pine) in which the interaction occurred.

The knowledge acquired is a crucial prerequisite for an effective habitat management program aimed at conserving the populations of these important predators.

## 2. Materials and Methods

### 2.1. Area of Study

The study was carried out in the Montes Orientales region, 20 km to the north of the Andalusian province of Granada, which is the fourth largest area devoted to olive grove crops, covering 198,331 hectares (ha) [81]. The landscape in this region is dominated by olive plantations, with patches of semi-natural vegetation mostly composed of *P. halepensis*, *Q. rotundifolia* and *P. dulcis*, in addition to less abundant species, such as *Quercus coccifera* L. (Fagales: Fagaceae), *Juniperus oxycedrus* L. (Pinales: Cupressaceae), *Cistus albidus* L. (Malvales: Cistaceae), *Cistus clusii* Dunal (Malvales: Cistaceae), *Genista cinerea* (Vill.) DC. (Fabales: Fabaceae), *Lavandula latifolia* Medik. (Lamiales: Lamiaceae), *Pistacia terebinthus* L. (Sapindales: Anacardiaceae), *Rosmarinus officinalis* L. (Lamiales: Lamiaceae), *Thymus mastichina* (L.) L. subsp. *mastichina* (Lamiales: Lamiaceae), *Thymus zygis* L. subsp. *gracilis* (Boiss) R. Morales (Lamiales: Lamiaceae) and *Ulex parviflorus* Pourr. (Fabales: Fabaceae).

Sampling was carried out in five organic olive farms (Table 1) in conformity with EU legislation [82,83]. All these farms are located at a similar altitude of 800 to 1100 m above sea level, the variety of *Olea europaea* L. is “Picual” and the plantation schemes are very similar (8 × 8 and 12 × 12 m), with areas ranging from 0.9 to 215 ha. Soil management practices on these farms include the maintenance of spontaneous vegetation cover, which is eliminated by mechanical mowing and/or grazing between April and May. In addition, during the post-harvest period, the soil is fertilized with organic matter, and crushed pruning waste is placed in the rows between crops to create inert cover. The incidence of disease (such as *Fusicladium oleagineum*) and pests (such as *P. oleae* and *Bactrocera oleae* (Gmelin, 1790)) was remedied by timely and targeted treatment (two aimed at diseases and one for pests) using products listed in Annex II of Commission Regulation (EC) no. 889/2008.

### 2.2. Collection of Samples

To collect the juvenile stages of chrysopids (larvae and prepupae/pupae), eight corrugated cardboard band traps (10 × 17.5 cm) were placed in a total of 100 trees (25 trees per species): *O. europaea* (olive), *Q. rotundifolia* (oak), *P. dulcis* (almond) and *P. halepensis* (pine), whose distribution in the sampling sites depended on their availability in the study area (Table 1). The band traps were installed on different branches located 160–170 cm from the ground taking into account the four cardinal directions (two band traps per direction). The 800 band traps were changed each month between June 2016 and May 2017 (a total of 12 sampling events) on the same 100 trees (identified by number).

In the laboratory, the juvenile stages—larvae, “open cocoons”, with one or more apertures caused by the emergence of chrysopid or parasitoid adults and predators feeding on juveniles, as well as “closed cocoons”, with no apertures and containing a chrysopid larva—were individually labelled and kept in Petri dishes (55 mm in diameter) for observation and monitoring. The trash-bearing juveniles (with exogenous material on their backs) and naked juveniles (with no exogenous material) were also quantified. The larval instars and “closed cocoons” were kept in an incubation chamber (Fitoclima S600 PLH; Aralab, Rio de Mouro, Portugal) in order to monitor their development at a temperature of 25 ± 1 °C, a humidity of 50%–60% and a photoperiod of 16:8 (Light:Dark) hours.

The individual larvae were fed *ad libitum* with *Ephestia kuehniella* Zeller (Lepidoptera: Pyralidae) eggs (EphestiaTop; Biotop; Livron-sur-Drôme; France) to facilitate the completion of their biological cycle up to the adult stage and taxonomic identification.

**Figure 1:**
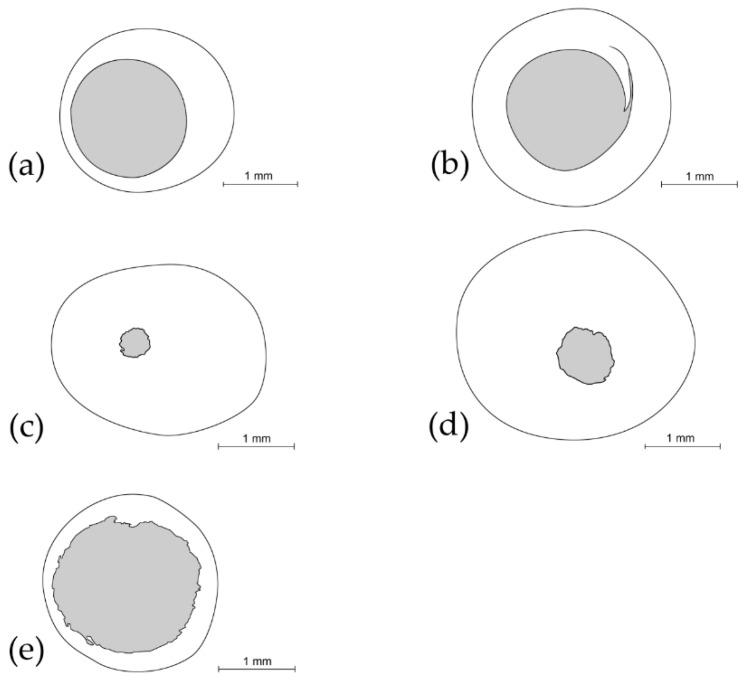
Examples of apertures in cocoons made by (**a**) the Chrysopidae family, by the most abundant parasitoid species: (**b**) *Helorus ruficornis*, (**c**) *Baryscapus impeditus* and (**d**) *Isodromus puncticeps* and by (**e**) predators.

The juveniles that failed to reach the adult stage were inspected under a stereomicroscope (Nikon SMZ 800; Nikon, Tokyo, Japan) in order to ascertain whether death was due to parasitoids or unknown factors. Additionally, we determined whether the aperture in the “open cocoons” was caused by the emergence of an adult chrysopid, a parasitoid or by the feeding of predators. In parasitized cocoons, the number of emerged adult parasitoids, as well as the number and average diameter of exit apertures were quantified (Figure 1).

The adult chrysopids that emerged in the laboratory were identified taxonomically up to species level according to the Monserrat key [1]. The emerged adult parasitoids in the laboratory were identified up to species level with the aid of taxonomists with specialist knowledge of the different families (see acknowledgements), the Plant Protection Group collection at the Estación Experimental del Zaidín (EEZ) and the Goulet and Huber key [84].

### 2.3. Statistical Analysis

All analyses were carried out using R software version 3.5.0 [85]. Statistical analysis began with data exploration [86]. We explored the total abundance of the juvenile stages collected in four categories (adult, parasitized, and predated chrysopids; unknown factors) in the tree species sampled throughout the study period. For data presentation purposes, the study period was simplified by grouping the sampling dates by season: Summer (June, July and August), autumn (September, October and November), winter (December, January and February) and spring (March, April and May). Juveniles (from larvae and “open or closed cocoons”), which produced an adult chrysopid and emerged either in the laboratory or in the field, were categorized under the heading “adult chrysopids”. A similar system was used for parasitoids from juveniles, which were grouped under the heading “parasitized chrysopids”. Death of juveniles caused by other population decline factors were classified as “unknown factors”. Finally, “open cocoons” with apertures due to attacks by predators, were defined as “predated chrysopids”.

We then analysed the total abundance of juvenile stages collected from each tree species sampled using a generalized linear mixed model (GLMM) with a negative binomial distribution (Equations (1)–(3)) and a log link function (Equation (4)) in relation to tree species, site and month sampled as fixed factors and the identification of the individual tree as the random factor (Equations (4) and (5)) using the “lme4” software package [87]:

Abundance of juvenile stages ~ NB(μ_ij_, k) (1)

E(Abundance of juvenile stages_ij_) = μ_ij_ (2)

(3) $$\mathrm{var}(\mathrm{Abundance}\;\mathrm{of}\;\mathrm{juvenile}\;{\mathrm{stages}}_{\mathrm{ij}})\;=\;\mu_{\mathrm{ij}}+\frac{\mu_{\mathrm{ij}}^{2}}{k}$$

Log(μ_ij_) = tree species_ij_+ site_ij_+ month sampled_ij_ + a_j_ (4)

a_j_ ~ N(0, σ^2^individual tree) (5)

We then calculated the rate of parasitism per tree (%) expressed as the number of juvenile stages affected by parasitism in each tree divided by the total number of juvenile stages collected from each tree multiplied by 100. The rate of parasitism was analysed with the aid of the GLMM with a binomial distribution (Equation (6)) and a logit link function (Equation (7)) using tree species, site and month sampled as fixed factors and the identification of the individual tree as the random factor (Equations (7) and (8)). The “lme4” software package was used for this analysis [87]:

Parasitism rate_ij_ ~ Bin(1, p_ij_) (6)

Logit(p_ij_) = α + β_1_ x Tree species_ij_+ β_2_ x site_ij_ + β_3_ x month sampled_ij_ + a_j_ (7)

a_j_ ~ N(0, σ^2^individual tree) (8)

The models were constructed and selected according to Akaike Information Criteria (AIC) [88]. We also analysed the model residuals and checked for uniformity using the “DHARMa” software package [89]. The multiple comparisons in each model (chrysopid abundance and parasitism rate) for the tree species, site and month sampled variables were checked with the aid of the post-hoc Tukey test using the “multcomp” software package [90].

The data for juveniles categorized as “unknown factors”, “predated chrysopids” and “adult chrysopids” were analysed by applying the Kruskal–Wallis test with a Bonferroni adjustment with the aid of the “agricolae” software package [91].

In addition, we calculated the parasitism rate according to the trash-bearing and naked juveniles collected. The rate of parasitism was analysed by applying the Kruskal–Wallis test with a Bonferroni adjustment with the aid of the “agricolae” software package [91].

We employed redundancy analysis (RDA) to determine whether a relationship exists between the composition of chrysopid and parasitoid species and environmental variables (tree species and season). The results were presented using a tri-plot correlation with the aid of the “vegan” software package [92].

## 3. Results

### 3.1. Analysis of Collected Cocoons

We separated the “open cocoons” from “closed cocoons”. “Open cocoons” were classified as “adult chrysopids” (Figure 1a) which emerged from a single circular orifice with a regular border and an average diameter of 1.65 ± 0.01 mm (*n* = 5 cocoon apertures). Parasitized juveniles were classified as “parasitized chrysopids” (Figure 1b–d) which emerged through one, two or three regular or irregular circular apertures with a diameter ranging from 0.4 to 1.7 mm (*n* = 15 cocoon apertures), with the remains of the juvenile host still inside the cocoon. “Open cocoons” were also classified as “predated chrysopids”, with one or two even or uneven circular apertures with an average diameter of 1.7 ± 0.07 mm (*n* = 5 cocoon apertures) (Figure 1e) to feed on juvenile stages, without remains of the juvenile host inside the cocoon. “Closed cocoons” contained prepupa or pupa which could emerge as “adult chrysopids”, could have become “parasitized chrysopids” or may not have emerged at all and died due to “unknown factors”.

A total of 1345 juvenile stages of chrysopids were collected between June 2016 and May 2017, over half of which (741 juveniles; *n* = 1200 trees sampled) completed their development to adulthood in the laboratory or in the field. The other juveniles (604 juveniles; *n* = 1200 trees sampled) failed to reach adulthood due to the action of parasitoids (357 juveniles; *n* = 1200 trees sampled), predators (69 juveniles; *n* = 1200 trees sampled) and unknown factors (178 juveniles; *n* = 1200 trees sampled) (Table 2).

### 3.2. Abundance and Identification of Chrysopids

The abundance of chrysopids fluctuated during all four seasons. According to the results of the GLMM (Table 3, Table S1), the summer months showed by far the greatest abundance of chrysopids per tree (2.58 ± 0.28; 774 juveniles; *n* = 300 trees sampled), while the winter months recorded the lowest abundance (0.23 ± 0.04; 68 juveniles; *n* = 300 trees sampled) (Table 2). The months of autumn (1.24 ± 0.14; 373 juveniles; *n* = 300 trees sampled) and spring (0.43 ± 0.06; 130 juveniles; *n* = 300 trees sampled) registered intermediate values. In the spring period, the abundance of juveniles in May (0.82 ± 0.16; *n* = 100 trees sampled) was higher than that in all the winter months: December (0.18 ± 0.05; *n* = 100 trees sampled), January (0.16 ± 0.05; *n* = 100 trees sampled) and February (0.33 ± 0.08; *n* = 100 trees sampled) (Table 3, Table S1).

Chrysopid abundance varied significantly between sites according to the GLMM (Table 3, Table S1); the Norberto farm presented the highest abundance (2.04 ± 0.25; *n* = 336 trees sampled) as compared to the other sites, while the Píñar farm (left) had the lowest abundance (0.47 ± 0.09; *n* = 216 trees sampled); the other sites (Los Almendros, Píñar farm (right) and La Pedriza) reported intermediate values and significant inter-site differences (Table 3, Table S1).

Tree species was also a variable factor in the abundance of juvenile stages of chrysopids (Table 3, Table S1). Pine trees exhibited significantly lower abundance of juveniles per tree (0.75 ± 0.13; 225 juveniles; *n* = 300 trees sampled) as compared to the other tree species: Almond (1.76 ± 0.27; 529 juveniles; *n* = 300 trees sampled), olive (1.19 ± 0.13; 356 juveniles; *n* = 300 trees sampled) and oak (0.78 ± 0.08; 235 juveniles; *n* = 300 trees sampled), with no significant differences being observed between the latter three species (Table 3, Table S1).

The number of juveniles that completed their development to adulthood was by far the highest for those sampled from olive trees (0.87 ± 0.1; 225 juveniles; *n* = 300 trees sampled) (Kruskal–Wallis χ² = 28.57, d.f. = 3, *p* < 0.001) and lowest in oak trees (0.4 ± 0.05; 121 juveniles; *n* = 300 trees sampled), with almond and pine trees recording intermediate values and with no significant differences between almond, oak and pine trees (Table 2). The number of juveniles killed by “unknown factors” was significantly higher in almond trees (0.22 ± 0.03; 66 juveniles; *n* = 300 trees sampled) than in oak (0.09 ± 0.02; 28 juveniles; n = 300 trees sampled) and pine trees (0.09 ± 0.03; 29 juveniles; *n* = 300 trees sampled) (Kruskal–Wallis χ² = 22.79, d.f. = 3, *p* < 0.001), while no significant differences were observed between almond, oak and pine trees, on the one hand, and olive trees (0.18 ± 0.04; 55 juveniles; *n* = 300 trees sampled), on the other (Table 2). Moreover, the number of “predated chrysopids” in all tree species studied did not differ significantly (Kruskal–Wallis χ² = 5.33, d.f. = 3, *p* = 0.15).

With regard to temporal distribution, the number of juveniles killed by “unknown factors” collected in summer (0.3 ± 0.05; 90 juveniles; *n* = 300 trees sampled) and autumn (0.17 ± 0.03; 51 juveniles; *n* = 300 trees sampled) was significantly higher than in spring (0.07 ± 0.03; 21 juveniles; n = 300 trees sampled) and winter (0.05 ± 0.02; 16 juveniles; *n* = 300 trees sampled), although no significant inter-seasonal differences were observed (Kruskal–Wallis χ² = 49.72,d.f. = 3, *p* < 0.001). The number of juveniles reaching adulthood was significantly higher in summer (1.37 ± 0.14; 410 juveniles; *n* = 300 trees sampled), followed by autumn (0.68 ± 0.08; 205 juveniles; *n* = 300 trees sampled), spring (0.29 ± 0.04; 86 juveniles; *n* = 300 trees sampled) and winter (0.13 ± 0.03; 40 juveniles; *n* = 300 trees sampled), with significant differences being observed between these last three seasons (Kruskal–Wallis χ² = 126.1, d.f. = 3, *p* < 0.001) (Table 2).

A total of 440 adult chrysopids belonging to ten species from five different genera of the family Chrysopidae emerged in the laboratory: *Chrysopa* Leach, 1815 (1), *Chrysoperla* (4), *Cunctochrysa* Hölzel, 1972 (1), *Pseudomallada* (3) and *Rexa* (1) (Table 4).

*Pseudomallada prasinus* (Burmeister, 1839) was the most abundant species (242 individuals) followed by *Chrysoperla pallida* Henry, Brooks, Duelli and Johnson, 2002 (74 individuals) and *Chrysoperla mediterranea* (Hölzel, 1972) (63 individuals). The other species were much less numerous: *Chrysoperla lucasina* (Lacroix, 1912) (16), *Chrysoperla mutata* (McLachlan, 1898) (15), *Rexa almerai* (Navás, 1919) (10), *Pseudomallada picteti* (McLachlan, 1880) (7), *Pseudomallada flavifrons* (Brauer, 1851) (5), *Chrysopa pallens* (Rambur, 1838) (4) and *Cunctochrysa baetica* (Hölzel, 1972) (4).

### 3.3. Parasitism Rate and Juvenile Chrysopid Parasitoid Complex

The rate of parasitism differed significantly in the arboreal stratum (Table 3, Table S2); the rate for olive trees (4.2 ± 1%; 28 parasitized juveniles; *n* = 300 trees sampled) was significantly below that for the other tree species: Almond trees (7.53 ± 1.23%; 199 parasitized juveniles; *n* = 300 trees sampled), oak trees (11.96 ± 1.66%; 74 parasitized juveniles; n = 300 trees sampled) and pine trees (6.54 ± 1.29%; 56 parasitized juveniles; *n* = 300 trees sampled); almond, oak and pine trees did not show any significant inter-species differences (Table 3, Table S2).

**Figure 2:**
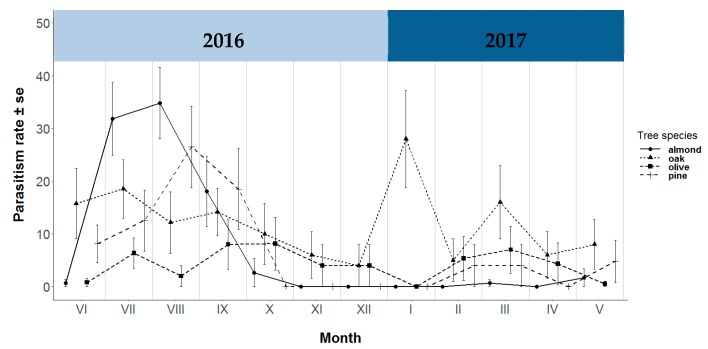
Temporal evolution of parasitism rate (%) in almond, oak, olive and pine trees by month sampled.

With regard to the temporal evolution of the parasitism rate, juvenile chrysopids collected in almond trees were found to be affected by parasitism between the months of July and September, reaching a maximum of 34.8% in August. A similar tendency was detected in pine trees, with a maximum of 26.5% recorded in August. On the other hand, juvenile chrysopids in olive and oak trees were affected by parasitism virtually throughout the whole period of the study, with oak trees displaying a maximum rate of 28% in January (Figure 2).

With respect to the sites sampled, the average rate of parasitism was found to be significantly higher in the Los Almendros farm (12.24 ± 2%; *n* = 216 trees sampled) as compared to the Norberto farm (8.49 ± 1.23%; *n* = 336 trees sampled), although differences in relation to the other farms (Píñar (right), La Pedriza and Píñar (left)) or with respect to inter-farm rates were not significant (Table 3, Table S2).

On the other hand, the parasitism rate of naked juveniles (5.08 ± 0.55%; 287 juveniles; *n* = 1200 trees sampled) was significantly higher than that for trash-bearing juveniles (3.69 ± 0.51%; 70 juveniles; *n* = 1200 trees sampled) (Kruskal–Wallis χ² = 11.64, d.f. = 1, *p* < 0.001).

A total of 1033 parasitoids belonging to five species from five different families of the Order Hymenoptera emerged in the laboratory from 174 parasitized juveniles: *Baryscapus impeditus* (Nees, 1834) (Chalcidoidea: Eulophidae), *Gelis ilicicola* (Seyrig, 1927) (Ichneumonoidea: Ichneumonidae), *Helorus ruficornis* Förster, 1856 (Proctotrupoidea: Heloridae), *Isodromus puncticeps* (Howard, 1885) (Chalcidoidea: Encyrtidae) and *Perilampus minutalis* Steffan, 1952 (Chalcidoidea: Perilampidae) (Table 5).

*Baryscapus impeditus* was the most numerous species (903 individuals from 84 parasitized juveniles). The number of parasitoids per parasitized juvenile ranged from one to 30 (10.75 ± 0.65; *n* = 84 parasitized juveniles), which emerged through one, two or three unevenly edged circular apertures with an average diameter of 0.42 ± 0.02 mm (*n* = 5 cocoon apertures) (Figure 1c). *Helorus ruficornis* was the second most abundant species (64 individuals from 64 parasitized chrysopids). A single parasitoid emerged from each cocoon through a single helicoidal-shaped aperture with a clearly defined edge and an average diameter of 1.72 ± 0.04 mm (*n* = 5 cocoon apertures) (Figure 1b). With respect to *Isodromus puncticeps* (52 individuals from 12 parasitized chrysopids), the number of individuals per parasitized chrysopid, which emerged, through a single unevenly edged circular aperture with an average diameter of 0.77 ± 0.04 mm (*n* = 5 cocoon apertures), ranged from one to ten (4.33 ± 0.85; *n* = 12 parasitized juveniles) (Figure 1d). The following species were much less abundant: Nine *Gelis iliciola* and five *Perilampus minutalis* individuals emerged through an unevenly edged aperture with a diameter of 1.11 ± 0.05 mm (*n* = 5 cocoon apertures) and 1.58 ± 0.26 mm (*n* = 5 cocoon apertures), respectively; in both species, each parasitoid emerged from a single parasitized juvenile.

### 3.4. Multivariate Analysis of the Relationship between Parasitoid and Chrysopid Species, Tree Species and Season

Using RDA analysis, we determined that tree species and season accounted for 14.1% of the variation in the parasitoid and chrysopid community. The first two RDA axes accounted for 79% of this variation and adjusted R² for 12.8%, suggesting that other variables were not captured by the model.

**Figure 3:**
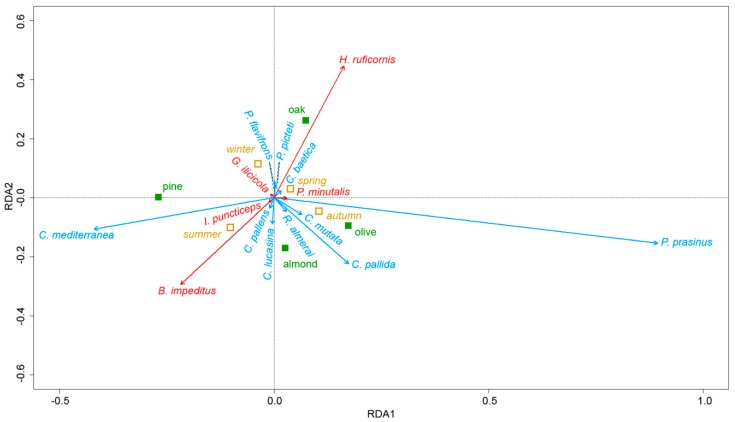
Redundancy analysis (RDA) tri-plot ordination showing variations in the abundance of the parasitoid complex (in red) and chrysopid species community (in blue) with respect to two nominal variables; tree species (in green) and season (in yellow).

The RDA correlation tri-plot (Figure 3) showed that three groups of species were positively inter-correlated. The first group was composed of three chrysopids (*C. baetica*, *P. flavifrons* and *P. picteti*) and one parasitoid (*H. ruficornis*). The abundance of *C. baetica* reached maximum levels in oak trees in autumn, with a similar pattern being observed for *P. flavifrons* and *P. picteti* only in spring, while the parasitoid *H. ruficornis* recorded maximum abundance in oak trees in all seasons (Table 4 and Table 5).

The second group was composed of three chrysopids (*C. pallens*, *C. lucasina* and *C. mediterranea*) collected in spring and summer and two parasitoids (*B. impeditus* and *I. puncticeps*) (Figure 3). *C. lucasina* appeared in spring in almond trees and then spread to the four tree species, while *C. pallens* was only detected in almond trees and *C. mediterranea* reached maximum abundance in pine trees in summer (Table 4 and Table 5). *B. impeditus* was mainly observed in almond trees and dispersed to pine trees in summer, though with a lower level of abundance, while the other parasitoid species *I. puncticeps* appeared in spring in almond and pine trees and had a preference for almond trees in summer (Table 4 and Table 5).

The third group is composed of *C. pallida, R. almerai*, *C. mutata*, *P. prasinus* and the parasitoid *P. minutalis*. *R. almerai* only appeared in olive trees in spring and summer, while *C. pallida* was reported in olive trees throughout the year, reaching maximum levels in almond trees in summer. *C. mutata* was mainly recorded in summer and autumn. Finally, *P. prasinus*, though collected from olive and almond trees throughout the year, reached maximum abundance in olive trees in autumn, with the parasitoid *P. minutalis* showing a similar pattern (Table 4 and Table 5).

## 4. Discussion

This study provides an insight into the abundance of chrysopid populations in olive groves, as well as almond, oak and pine trees adjacent to the crop, in addition to population decline factors. Juvenile stages of chrysopids were more abundant in almond, oak and olive trees than in pine trees. We found that parasitoids and chrysopids shared a similar temporal pattern in our study area. Additionally, the period of parasitoid incidence was found to extend beyond the April to November period previously reported [78,93]. We observed that parasitoid abundance was highest in the summer months in olive trees, which is in line with the findings of Neuenschwander and Michelakis [80] and Campos [78].

The presence of “predated chrysopids” and “unknown factors” had a marked seasonal character, with the largest number in both categories recorded in summer, when the environment is less humid and temperatures are higher than in other seasons. This concurs with the results of previous studies which demonstrate that conditions, such as low humidity and high temperatures lead to increased mortality and slower development in the preimaginal stages [35,59,94]. This slower development could also render the juvenile stages more vulnerable to predators.

Overall, we found that mortality caused by parasitism (26.5%) constitutes a major chrysopid population decline factor. Although this is very similar to the level (27.7%) determined by Campos [78] in olive groves in southern Spain, it is quite low compared to the levels (80% and 54.9%, respectively) reported in olive groves by Alrouechdi et al. [50] in France and Neuenschwander and Michelakis [80] in Crete.

With regard to tree species, the parasitism rate per tree in olive trees was very low as compared to previous studies [50,78,80,93] and considerably lower than that in the three arboreal species (almond, oak and pine) studied. This, together with predation and unknown factors, make olive trees the most important arboreal species with regard to the number of viable next-generation adult chrysopids.

The highest rate of parasitism recorded in almond, oak and pine trees could be due to their location in semi-natural areas bordering the crop. The semi-natural habitats and landscape bordering the crop are characterized by greater species richness and parasitoid diversity than other types of habitat such as crop and vegetation cover [95]. Few data are available on the seasonality of parasitism in these trees. However, we demonstrated that the parasitism rate in pine and almond trees is higher in the summer months, which is similar to the pattern found by Judd [58] in pine trees. Oak trees showed a more-or-less constant rate of parasitism throughout the year, which is similar to the rate of close to 15% recorded in other studies [96]. Additionally, oak trees become a parasitoid bank in winter due to their high rate of parasitism. This could have a negative effect on the next chrysopid generation and enable parasitoids to move into olive groves in spring. However, low rates of parasitism in olive trees and high rates in oak trees in spring suggest that parasitoids remain in oak trees. As almond trees have a high rate of parasitism in summer and are a good reservoir of juvenile chrysopids, they could play an important role in increasing chrysopid populations in olive groves in the summer months, when *P. oleae* are especially harmful to olive trees.

The chrysopid community is composed of ten species in our biotope, with, as already noted in previous studies, *P. prasinus* and the *C. carnea* complex accounting for the majority of individuals [21,29,97]. On the other hand, studies focusing on the parasitoid complex of chrysopids have reported that a relationship exists between chrysopid species and their associated parasitoids [45,49,56]. The parasitoid complex is composed of five species: Three primary parasitoids (*B. impeditus*, *H. ruficornis* and *I. puncticeps*), with the highest levels of abundance, and two primary parasitoids, which also could act as hyperparasitoids (*G. ilicicola* and *P. minutalis*), with the lowest levels of abundance.

*B. impeditus*, the most abundant species, affected a large number of chrysopids, mainly juveniles of the species *C. mediterranea*, *C. lucasina* and *C. pallens*, which were collected in almond and pine trees. Our results regarding this parasitoid, which is characterized by gregarious behaviour and emerges from the host through various orifices, corroborate the findings of previous studies [45,50]. Although the period of activity of *B. impeditus* was similar to that in olive groves in Crete and France, the number of parasitoids per host was larger in our study [47,80].

The second most important parasitoid was *H. ruficornis*, which is found in Palearctic, Nearctic and Afrotropical regions [98,99,100]. This species has been previously cited in the Iberian Peninsula [101], specifically in olive groves [78,93]. Our findings would appear to contradict those of New [56], who has stated that *H. ruficornis* is in a minority among species in the chrysopid parasitoid complex in Europe due to competition from other parasitoids for hosts. In our study, the second most abundant parasitoid *H. ruficornis*, which competed with four parasitoid species, plays a similar role to that observed by New [56]. Although little is known about its biology, *H. ruficornis* can, in our view, be classified as a solitary parasitoid, as only one parasitoid exits in the host cocoon. This behaviour resembles that of other species of the same genus and concurs with other studies which suggest that all species of the genus *Helorus* are biologically similar [45,48,51,56,98]. *H. ruficornis* has also been shown to parasitize species of the genera *Chrysoperla*, *Pseudomallada*, *Chrysopa*, and *Nineta* [45,46,51,56]. We observed that *H. ruficornis* parasitizes the juvenile stages of the genera *Pseudomallada* (*P. picteti*, *P. flavifrons* and *P. prasinus*) and *C. baetica* which have a preference for oak trees in the Iberian Peninsula [21,102].

Of the two species from the genus *Isodromus* that parasitize chrysopids [48], we collected *I. puncticeps*, which is in a minority in the parasitoid complex studied. Although this resembles the pattern observed in Greek olive groves [56,78,80,96], *I. puncticeps* plays an important role in French olive groves [47,50,103]. With the aid of RDA analysis, although we found a positive relationship between the abundance of *B. impeditus* and *I. puncticeps*, given the insufficient number of individuals of the latter, we were unable to shed any light on this relationship. Nevertheless, as previously described by Clancy [45] and Campos [78], we found *I. puncticeps* to be a gregarious parasitoid.

While the characteristics that enable chrysopids to protect against natural enemies include the use of exogenous trash by juveniles as a defensive shield against predation [72], evidence with regard to parasitism is less clear [49,71,104]. In our study, the rate of parasitism was found to be higher in naked chrysopid species (*C. lucasina*, *C. mediterranea*, *C. mutata*, *C. pallida* and *C. pallens*) as compared to trash-bearing species (*C. baetica*, *P. flavifrons*, *P. picteti*, *P. prasinus* and *R. almerai*); however Muma [49] found that the rate of parasitism is lower in naked chrysopids than in more abundant trash-bearing chrysopids. Therefore, depending on chrysopid assemblage and abundance, as well as the parasitoid complex associated with each geographical area, rates of parasitism will, in our view, be affected by whether juvenile chrysopids are trash-bearing or naked. However further research is required to cast light on this relationship.

## 5. Conclusions

We have demonstrated that chrysopid abundance in almond and oak tree species in the arboreal stratum adjacent to olive groves is comparable to that in olive trees. With regard to population dynamics, the combined effect of three decline factors (parasitism, predation and unknown factors) of chrysopid populations over the short term needs to be taken into account when habitat management is being considered to conserve these populations. Additionally, in the biotope studied, we found that ten chrysopid species use the arboreal stratum to develop their biological cycle, in which *P. prasinus* is the most abundant species. We also found that three out of the five species in the parasitoid complex of the family Chrysopidae are primary parasitoids, with *B. impeditus* showing a preference for *C. pallens*, *C. lucasina* and *C. mediterranea*; and *H. ruficornis* being associated with *C. baetica*, *P. flavifrons* and *P. picteti*, representing the majority of parasitoid species. A knowledge of chrysopid population decline factors in semi-natural habitats could be crucial for an effective habitat management program aimed at conserving and expanding chrysopid populations to boost the presence of chrysopids and the natural pressure on pests and to contribute to olive grove sustainability.

## Figures and Tables

**Table 1:** Characteristics and availability of each tree species and number of tree species sampled in each site per month sampled.

Site	Coordinates	Area (ha)	Number of Trees Sampled				
			Almond	Oak	Olive	Pine	Total
Norberto	37°19’5.96” N; 3°34’9.92” W	4.3	9	5	5	9	28
La Pedriza	37°20’17.44” N; 3°33’39.21” W	0.9	-	5	5	8	18
Los Almendros	37°22’24.76” N; 3°37’46.03” W	215	8	5	5	-	18
Píñar (right)	37°24’14.29” N; 3°29’14.13” W	58	-	5	5	8	18
Píñar (left)	37°24’40.93” N; 3°28’52.41” W	124	8	5	5	-	18
Total			25	25	25	25	100

**Table 2:** Abundance (%) and categories of juvenile stages in almond, oak, olive and pine trees by season.

Season	Tree Species	Adult Chrysopids	Parasitized Chrysopids	Predated Chrysopids	Unknown Factors	Total
Summer	Almond	122 (36.7)	144 (43.4)	33 (9.9)	33 (9.9)	332
	Oak	49 (48)	34 (33.3)	9 (8.8)	10 (9.8)	102
	Olive	130 (76.9)	9 (5.3)	8 (4.7)	22 (13)	169
	Pine	109 (63.7)	34 (19.9)	3 (1.8)	25 (14.6)	171
	Subtotal	410 (53)	221 (28.6)	53 (6.8)	90 (11.6)	774
Autumn	Almond	67 (45.3)	54 (36.5)	6 (4.1)	21 (14.2)	148
	Oak	43 (55.8)	18 (23.4)	3 (3.9)	13 (16.9)	77
	Olive	77 (72.6)	11 (10.4)	4 (3.8)	14 (13.2)	106
	Pine	18 (42.9)	19 (45.2)	2 (4.8)	3 (7.1)	42
	Subtotal	205 (55)	102 (27.3)	15 (4)	51 (13.7)	373
Winter	Almond	13 (68.4)	0 (0)	0 (0)	6 (31.6)	19
	Oak	3 (18.8)	11 (68.8)	0 (0)	2 (12.5)	16
	Olive	24 (72.7)	1 (3)	0 (0)	8 (24.2)	33
	Pine	0 (0)	0 (0)	0 (0)	0 (0)	0
	Subtotal	40 (58.8)	12 (17.6)	0 (0)	16 (23.5)	68
Spring	Almond	23 (76.7)	1 (3.3)	0 (0)	6 (20)	30
	Oak	26 (65)	11 (27.5)	0 (0)	3 (7.5)	40
	Olive	29 (60.4)	7 (14.6)	1 (2.1)	11 (22.9)	48
	Pine	8 (66.7)	3 (25)	0 (0)	1 (8.3)	12
	Subtotal	86 (66.2)	22 (16.9)	1 (0.8)	21 (16.2)	130
Total		741 (55.1)	357 (26.5)	69 (5.1)	178 (13.2)	1345

**Table 3:** ANOVA (type II Wald Chi-square test) results of generalized linear mixed models (GLMMs) (chrysopid abundance and parasitism rate). Significance codes: *** *p* < 0.001, ** *p* < 0.01, * *p* < 0.05.

Model	Variable	χ²	Degree of Freedom (d.f.)	*p* Value	
Chrysopid abundance	Tree species	29.168	3	<0.001	***
	Site	48.165	4	<0.001	***
	Month sampled	320.795	11	<0.001	***
Parasitism rate	Tree species	34.707	3	<0.001	***
	Site	11.832	4	0.0187	*
	Month sampled	57.895	11	<0.001	***

**Table 4:** Abundance (mean ± SE) of chrysopid species that emerged in laboratory from chrysopid juveniles collected from almond, oak, olive and pine trees by season.

Season	Tree Species	*Cunctochrysa baetica*	*Chrysoperla lucasina*	*Chrysoperla mediterranea*	*Chrysoperla mutata*	*Chrysoperla pallida*	*Chrysopa pallens*	*Pseudomallada flavifrons*	*Pseudomallada picteti*	*Pseudomallada prasinus*	*Rexa almerai*
Summer	Almond	*	0.04 ± 0.02	0	0.04 ± 0.02	0.36 ± 0.09	0.04 ± 0.02	0	*	0.41 ± 0.13	0
	Oak	*	*	0	0.04 ± 0.02	0.07 ± 0.03	0	0	0	0.32 ± 0.08	0
	Olive	0	0.05 ± 0.03	*	0.05 ± 0.03	0.28 ± 0.1	0	0	0	0.12 ± 0.04	0.05 ± 0.03
	Pine	0	0.03 ± 0.02	0.52 ± 0.25	0	0	0	0	0	0	0
Autumn	Almond	0	0	*	0	*	0	0	0	0.45 ± 0.14	0
	Oak	0.03 ± 0.02	0	0	*	0	0	0	*	0.17 ± 0.05	0
	Olive	0	0	0	0.04 ± 0.02	0.12 ± 0.04	0	0	*	0.52 ± 0.1	0
	Pine	0	0	0	0	0	0	0	*	*	0
Winter	Almond	0	0	0	0	0	0	0	0	0.15 ± 0.05	0
	Oak	0	0	0	0	0	0	*	0	0.09 ± 0.05	0
	Olive	0	0	0	*	*	0	0	0	0.25 ± 0.08	0
	Pine	0	0	0	0	0	0	0	0	*	0
Spring	Almond	0	0.08 ± 0.04	0	0	*	*	0	0	0.25 ± 0.1	0
	Oak	0	0	0	0	0.07 ± 0.03	0	0.04 ± 0.02	0.03 ± 0.02	0.13 ± 0.04	0
	Olive	0	0	0	0	0.05 ± 0.04	0	0	0	0.31 ± 0.08	0.08 ± 0.06
	Pine	0	0	0.29 ± 0.12	0	0	0	*	*	*	0

* Mean ± standard error ≤ 0.01 ± 0.01.

**Table 5:** Abundance of juvenile chrysopids parasitized (mean ± SE) by the parasitoid species complex in almond, oak, olive and pine trees by season.

Season	Tree Species	Juvenile Chrysopids Parasitized by				
		*Baryscapus impeditus*	*Gelis ilicicola*	*Helorus ruficornis*	*Isodromus puncticeps*	*Perilampus minutalis*
Summer	Almond	0.88 ± 0.23	0.03 ± 0.02	0	0.03 ± 0.02	0
	Oak	0	0.05 ± 0.03	0.16 ± 0.05	*	0
	Olive	0	*	*	*	0
	Pine	0.15 ± 0.05	0	0.11 ± 0.05	0	0
Autumn	Almond	*	0	*	*	0
	Oak	0	0	0.17 ± 0.05	0	0
	Olive	*	0	0.07 ± 0.04	0	0.03 ± 0.02
	Pine	0.05 ± 0.04	0	0	0	0
Winter	Almond	0	0	0	0	0
	Oak	0	0	0.15 ± 0.05	0	0
	Olive	0	0	0.04 ± 0.02	0	0
	Pine	0	*	0	0	0
Spring	Almond	0	0	0	0.04 ± 0.04	*
	Oak	*	0	0.09 ± 0.04	0.04 ± 0.02	*
	Olive	0	*	0.04 ± 0.02	0	*
	Pine	0	0	0	*	0

* Mean ± standard error ≤ 0.01 ± 0.01.

## Supplementary Materials

The following are available online at https://www.mdpi.com/2075-4450/10/5/134/s1, Table S1: Multiple comparisons of generalized linear mixed model (GLMM) abundance of juvenile stages of chrysopids in relation to tree species, site and month sampled including estimate, standard error (SE) and *p* value. Significance codes: *** *p* < 0.001, ** *p* < 0.01, * *p* < 0.05, Table S2: Multiple comparisons of GLMM parasitism in relation to tree species, site and month sampled including estimate, standard error (SE) and *p* value. Significance codes: *** *p* < 0.001, ** *p* < 0.01, * *p* < 0.05.
