# Supplementary material for: Abundance and Population Decline Factors of Chrysopid Juveniles in Olive Groves and Adjacent Trees
Source: Insects. 2019 May 7;10(5):134. doi: 10.3390/insects10050134 (PMC6572422; doi:10.3390/insects10050134)
Supplement: Supplementary file 1 [file insects-10-00134-s001.pdf]

Table S1. Multiple comparisons of generalized linear mixed model (GLMM) abundance of juvenile stages of chrysopids in relation to tree species, site and month sampled including estimate, standard error (SE) and *p* value. Significance codes: *** $p < 0.001$, ** $p < 0.01$, * $p < 0.05$.

Variable	Comparison	Estimate	SE	*p* Value	
Tree species	oak-almond	−0.1767	0.2287	0.86647	
	olive-almond	0.2701	0.2288	0.63853	
	pine-almond	−0.9539	0.2512	<0.001	***
	olive-oak	0.4467	0.2156	0.16183	
	pine-oak	−0.7772	0.2363	0.00552	**
	pine-olive	−1.2239	0.2310	<0.001	***
Site	Norberto—Los Almendros	0.84625	0.23155	0.00229	**
	Píñar (right)—Los Almendros	0.05453	0.27889	0.99967	
	La Pedriza—Los Almendros	0.09863	0.28184	0.99673	
	Píñar (left)—Los Almendros	−0.84413	0.26956	0.01456	*
	Píñar (right)—Norberto	−0.79172	0.23892	0.00804	**
	La Pedriza—Norberto	−0.74763	0.24434	0.01856	*
	Píñar (left)—Norberto	−1.69039	0.25117	<0.001	***
	La Pedriza—Píñar (right)	0.04410	0.26624	0.99983	
	Píñar (left)—Píñar (right)	−0.89866	0.28928	0.01587	*
	Píñar (left)—La Pedriza	−0.94276	0.29044	0.01012	*
Month sampled	July 16–June16	0.033	0.19921	1	
	August 16–June16	−0.04597	0.20137	1	
	September 16–June16	−0.6002	0.21017	0.1487	
	October 16–June16	−1.575	0.23557	<0.001	***
	November 16–June16	−2.06946	0.25898	<0.001	***
	December 16–June 16	−2.83195	0.31197	<0.001	***
	January 17–June 16	−2.84347	0.32068	<0.001	***
	February 17–June 16	−2.12561	0.2646	<0.001	***
	March 17–June 16	−1.64999	0.24037	<0.001	***
	April 17–June 16	−1.96602	0.26099	<0.001	***
	May 17–June 16	−1.15665	0.22479	<0.001	***
	August 16–July 16	−0.07897	0.19722	1	
	September 16–July 16	−0.6332	0.20585	0.0834	
	October 16–July 16	−1.60801	0.23308	<0.001	***
	November 16–July 16	−2.10246	0.25581	<0.001	***
	December 16–July 16	−2.86495	0.30851	<0.001	***
	January 17–July 16	−2.87647	0.31837	<0.001	***
	February 17–July 16	−2.15861	0.26253	<0.001	***
	March 17–July 16	−1.68299	0.23745	<0.001	***
	April 17–July 16	−1.99902	0.25908	<0.001	***
	May 17–July 16	−1.18965	0.22219	<0.001	***
	September 16–August 16	−0.55423	0.20675	0.2254	
	October 16–August 16	−1.52903	0.23467	<0.001	***
	November 16–August 16	−2.02348	0.2578	<0.001	***
	December 16–August 16	−2.78598	0.30987	<0.001	***
	January 17–August 16	−2.7975	0.31968	<0.001	***
	February 17–August 16	−2.07964	0.26363	<0.001	***
	March 17–August 16	−1.60402	0.23885	<0.001	***
	April 17–August 16	−1.92005	0.2609	<0.001	***
	May 17–August 16	−1.11068	0.22364	<0.001	***
	October 16–September 16	−0.97481	0.24036	<0.01	**
	November 16–September 16	−1.46926	0.26279	<0.001	***
	December 16–September 16	−2.23175	0.31469	<0.001	***
	January 17–September 16	−2.24327	0.32403	<0.001	***

Continued Table S1

Variable	Comparison	Estimate	SE	*p* Value	
Month sampled	February 17–September 16	−1.52541	0.26859	<0.001	***
	March 17–September 16	−1.04979	0.24456	<0.01	**
	April 17–September 16	−1.36583	0.26546	<0.001	***
	May 17–September 16	−0.55645	0.23184	0.3909	
	November 16–October 16	−0.49445	0.28263	0.8363	
	December 16–October 16	−1.25694	0.33191	<0.01	**
	January 17–October 16	−1.26847	0.34036	<0.01	**
	February 17–October 16	−0.55061	0.28825	0.7414	
	March 17–October 16	−0.07499	0.2664	1	
	April 17–October 16	−0.39102	0.28653	0.9676	
	May 17–October 16	0.41835	0.25561	0.8892	
	December 16–November 16	−0.76249	0.34762	0.5408	
	January 17–November 16	−0.77402	0.3566	0.557	
	February 17–November 16	−0.056 16	0.30775	1	
	March 17–November 16	0.41946	0.28748	0.9478	
	April 17–November 16	0.10343	0.30481	1	
	May 17–November 16	0.9128	0.27685	0.0429	*
	January 17–December 16	−0.01152	0.39627	1	
	February 17–December 16	0.70634	0.35289	0.6792	
	March 17–December 16	1.18196	0.33567	0.0197	*
	April 17–December 16	0.86593	0.3505	0.3438	
	May 17–December 16	1.6753	0.32662	<0.001	***
	February 17–January 17	0.71786	0.3612	0.6899	
	March 17–January 17	1.19348	0.34413	0.0252	*
	April 17–January 17	0.87745	0.35842	0.3598	
	May 17–January 17	1.68682	0.33512	<0.001	***
	March 17–February 17	0.47562	0.29242	0.893	
	April 17–February 17	0.15959	0.31026	1	
	May 17–February 17	0.96896	0.28231	0.0285	*
	April 17–March 17	−0.31603	0.29025	0.9947	
	May 17–March 17	0.49334	0.25982	0.7488	
	May 17–April 17	0.80937	0.27614	0.1226	

Table S2. Multiple comparisons of GLMM parasitism in relation to tree species, site and month sampled including estimate, standard error (SE) and *p* value. Significance codes: *** $p < 0.001$, ** $p < 0.01$, * $p < 0.05$.

Variable	Comparison	Estimate	SE	*p* Value	
Tree species	oak-almond	−0.2526	0.2587	0.761	
	olive-almond	−1.7424	0.3189	<0.001	***
	pine-almond	−0.2108	0.2827	0.878	
	olive-oak	−1.4897	0.3016	<0.001	***
	pine-oak	0.0418	0.2926	0.999	
	pine-olive	1.5315	0.3253	<0.001	***
Site	Norberto—Los Almendros	−0.71149	0.24846	0.0322	*
	Píñar (right)—Los Almendros	−0.22743	0.33585	0.9594	
	La Pedriza—Los Almendros	−0.65332	0.37438	0.3941	
	Píñar (left)—Los Almendros	−0.99089	0.42913	0.1351	
	Píñar (right)—Norberto	0.48406	0.29848	0.4709	
	La Pedriza—Norberto	0.05818	0.33984	0.9998	
	Píñar (left)—Norberto	−0.27939	0.41113	0.9589	
	La Pedriza—Píñar (right)	−0.42588	0.3692	0.7692	
	Píñar (left)—Píñar (right)	−0.76346	0.4485	0.4202	
	Píñar (left)—La Pedriza	−0.33757	0.47338	0.9512	

Continued Table S2

Variable	Comparison	Estimate	SE	*p* Value	
	July 16–June 16	1.49404	0.25556	<0.001	***
	August 16–June 16	1.18344	0.26201	<0.001	***
	September 16–June 16	1.10039	0.29752	<0.001	***
	October 16–June 16	0.82897	0.42708	0.6854	
	November 16–June 16	−0.12214	0.66692	1	
	December 16–June 16	0.08032	0.8091	1	
	January 17–June 16	2.0855	0.60708	0.0232	*
	February 17–June 16	0.50792	0.56622	0.9988	
	March 17–June 16	1.04374	0.41918	0.2975	
	April 17–June 16	0.38706	0.56283	0.9999	
	May 17–June 16	−0.20285	0.42731	1	
	August 16–July 16	−0.3106	0.18579	0.8516	
	September 16–July 16	−0.39365	0.236	0.8536	
	October 16–July 16	−0.66508	0.38744	0.8279	
	November 16–July 16	−1.61618	0.64051	0.2784	
	December 16–July 16	−1.41372	0.78589	0.7799	
	January 17–July 16	0.59146	0.58136	0.9962	
	February 17–July 16	−0.98612	0.54068	0.7648	
	March 17–July 16	−0.4503	0.37874	0.9859	
	April 17–July 16	−1.10698	0.53901	0.6033	
	May 17–July 16	−1.69689	0.39098	<0.001	***
	September 16–August 16	−0.08306	0.23919	1	
	October 16–August 16	−0.35448	0.39136	0.9987	
Month sampled	November 16–August 16	−1.30558	0.64298	0.6202	
	December 16–August 16	−1.10312	0.78731	0.9516	
	January 17–August 16	0.90206	0.58366	0.9071	
	February 17–August 16	−0.67552	0.54107	0.9794	
	March 17–August 16	−0.1397	0.38336	1	
	April 17–August 16	−0.79639	0.54061	0.932	
	May 17–August 16	−1.38629	0.393	0.0165	*
	October 16–September 16	−0.27142	0.41138	0.9999	
	November 16–September 16	−1.22252	0.65552	0.7376	
	December 16–September 16	−1.02007	0.79952	0.9756	
	January 17–September 16	0.98512	0.59615	0.8611	
	February 17–September 16	−0.59247	0.557	0.9944	
	March 17–September 16	−0.05665	0.40542	1	
	April 17–September 16	−0.71333	0.55514	0.9743	
	May 17–September 16	−1.30323	0.42058	0.0666	
	November 16–October 16	−0.9511	0.72103	0.9687	
	December 16–October 16	−0.74864	0.85499	0.999	
	January 17–October 16	1.25654	0.66456	0.721	
	February 17–October 16	−0.32105	0.62972	1	
	March 17–October 16	0.21478	0.50389	1	
	April 17–October 16	−0.44191	0.63352	0.9999	
	May 17–October 16	−1.03181	0.52158	0.6584	
	December 16–November 16	0.20246	0.99469	1	
	January 17–November 16	2.20764	0.83912	0.2222	

Continued Table S2

Variable	Comparison	Estimate	SE	*p* Value	
	February 17–November 16	0.63006	0.8147	0.9997	
	March 17–November 16	1.16588	0.71993	0.8767	
	April 17–November 16	0.50919	0.81172	1	
	May 17–November 16	−0.08071	0.73	1	
	January 17–December 16	2.00518	0.95658	0.5715	
	February 17–December 16	0.4276	0.93399	1	
	March 17–December 16	0.96342	0.85254	0.9908	
	April 17–December 16	0.30674	0.93292	1	
	May 17–December 16	−0.28317	0.86282	1	
Month sampled	February 17–January 17	−1.57758	0.76314	0.5935	
	March 17–January 17	−1.04176	0.66276	0.897	
	April 17–January 17	−1.69845	0.75913	0.4673	
	May 17–January 17	−2.28835	0.67734	0.0276	*
	March 17–February 17	0.53582	0.62987	0.9993	
	April 17–February 17	−0.12086	0.73481	1	
	May 17–February 17	−0.71077	0.64119	0.9921	
	April 17–March 17	−0.65668	0.62701	0.9951	
	May 17–March 17	−1.24659	0.51427	0.3389	
	May 17–April 17	−0.5899	0.63654	0.9984	
